# Long-term outcomes for neoadjuvant versus adjuvant chemotherapy in operable breast cancer patients with hormone receptor-positive, HER2-negative

**DOI:** 10.3389/fonc.2026.1826224

**Published:** 2026-05-08

**Authors:** Yirui Wei, Mengyuan Wang, Yong Huang, Lu Gan, Kangjie Li, Yifeng Li, Zhaoyang Li, Qiao Cheng

**Affiliations:** 1 Department of Breast and Thyroid Surgery, The First Affiliated Hospital of Chongqing Medical University, Chongqing, China; 2 Breast Center, Chongqing University Three Gorges Hospital, Chongqing, China; 3 Department of Oncology, The First Affiliated Hospital of Chongqing Medical University, Chongqing, China; 4 College of Public Health, Chongqing Medical University, Chongqing, China

**Keywords:** breast neoplasms, chemotherapy, adjuvant, neoadjuvant therapy, receptors, estrogen, SEER program, survival analysis

## Abstract

**Objective:**

To compare the long-term survival outcomes of patients with operable hormone receptor-positive, human epidermal growth factor receptor 2-negative (HR+/HER2-) breast cancer at stage II and T3N1M0 receiving neoadjuvant chemotherapy (NACT) versus adjuvant chemotherapy (ACT) using data extracted from the Surveillance, Epidemiology, and End Results (SEER) database.

**Methods:**

Data on 33,552 patients were extracted from the SEER database between 2010 and 2020. Patients were categorized into the NACT group and the ACT group. The primary endpoints were overall survival (OS) and breast cancer-specific survival (BCSS). After propensity score matching (PSM), Kaplan-Meier survival analysis and Cox proportional hazards regression models were used to assess the effects of NACT versus ACT on survival outcomes. Subgroup analysis was performed to explore the impact of various clinical and pathological factors on survival outcomes.

**Results:**

After PSM, the NACT group showed significantly inferior 5-year OS (87.4% vs 91.8%, P<0.0001) and BCSS (89.3% vs 93.7%, P<0.0001) compared to the ACT group. Even among patients achieving objective response to NACT, multivariate Cox regression demonstrated significantly higher risks of breast cancer-specific death (HR = 1.42, 95%CI: 1.17-1.73, P<0.001) and all-cause death (HR = 1.31, 95%CI: 1.10-1.57, P = 0.002) versus the ACT group.

**Conclusion:**

In patients with operable HR+/HER2- breast cancer at stage II and T3N1M0, NACT was associated with inferior OS and BCSS compared to ACT. These findings suggest caution when selecting NACT for this patient subset.

## Introduction

Neoadjuvant chemotherapy (NACT) has emerged as an important treatment strategy for breast cancer. Landmark trials, including the National Surgical Adjuvant Breast and Bowel Project (NSABP) B-18 and B-27, along with the 2018 Early Breast Cancer Trialists’ Collaborative Group (EBCTCG) meta-analysis, have established that NACT and adjuvant chemotherapy (ACT) yield equivalent survival outcomes (1, 2). Furthermore, NACT offers additional advantages, including tumor downstaging to facilitate breast-conserving surgery (BCS) or axillary conservation, as well as providing *in vivo* assessment of chemotherapy sensitivity to guide postoperative treatment decisions (3). These benefits are particularly pronounced in triple-negative breast cancer (TNBC) and human epidermal growth factor receptor 2-positive (HER2+) breast cancer (4, 5).

For hormone receptor-positive, HER2-negative (HR+/HER2-) breast cancer, the pathological complete response (pCR) rate remains low, typically ranging from 5% to 15%, owing to the inherent insensitivity of this subtype to cytotoxic chemotherapy (6–8). Moreover, unlike in TNBC and HER2+ subtypes, achieving pCR in HR+/HER2- breast cancer demonstrates only a weak correlation with event-free survival (EFS) and overall survival (OS) (9, 10). Currently, international guidelines regarding the indications for NACT in this subgroup remain inconsistent. The Chinese Society of Clinical Oncology (CSCO) breast cancer guidelines recommend considering NACT for patients with tumors larger than T2 or lymph node involvement of N1 or greater (11). The National Comprehensive Cancer Network (NCCN) Clinical Practice Guidelines in Oncology indicate that preoperative systemic therapy may be preferred for patients with clinically node-positive (cN+) disease who may be downstaged to clinically node-negative (cN0) status following treatment (12). The China Anti-Cancer Association Committee of Breast Cancer Society (CACA-CBCS) guidelines suggest that patients with operable HR+/HER2- breast cancer should proceed directly to surgery unless downstaging for BCS or axillary conservation is required (13). The American Society of Clinical Oncology (ASCO) Clinical Practice Guidelines indicate that NACT may be considered in patients with HR+/HER2- tumors when treatment decisions can be made without surgical pathology information (5). The European Society for Medical Oncology (ESMO) Clinical Practice Guidelines recommend NACT for patients with large tumors or clinical lymph node involvement (14).

A recent study reported that among patients with T2N1M0 HR+/HER2- breast cancer, OS and breast cancer-specific survival (BCSS) were inferior in the NACT group compared with the ACT group (15). However, this study was limited to the T2N1M0 stage, which does not represent the only subgroup with clinical uncertainty regarding optimal treatment sequencing. In contrast, a Swedish population-based cohort study including 14,459 patients with operable HR+/HER2- breast cancer demonstrated comparable long-term survival outcomes between patients receiving NACT and those receiving ACT (16).

Therefore, this retrospective cohort study utilized data from the Surveillance, Epidemiology, and End Results (SEER) database to compare the long-term survival outcomes of NACT versus ACT in patients with HR+/HER2- breast cancer at stage II and T3N1M0. The objective was to determine the optimal chemotherapy sequencing strategy for this population and to provide evidence for clinical decision-making and future research.

## Methods

### Data source and study population

This retrospective cohort study was conducted using data from the Surveillance, Epidemiology, and End Results (SEER) database (SEER*Stat 8.4.3), which is a population-based cancer registry representing approximately 48% of the U.S. population. Data on patients diagnosed with HR+/HER2- breast cancer between 2010 and 2020 were retrieved from 17 registries released in April 2023. HR+ was defined as ≥1% of tumor cells staining positively for estrogen receptor or progesterone receptor, and HER2- was defined as immunohistochemistry staining of 0 or 1+, or 2+ with fluorescence *in situ* hybridization-negative results.

**Figure 1:**
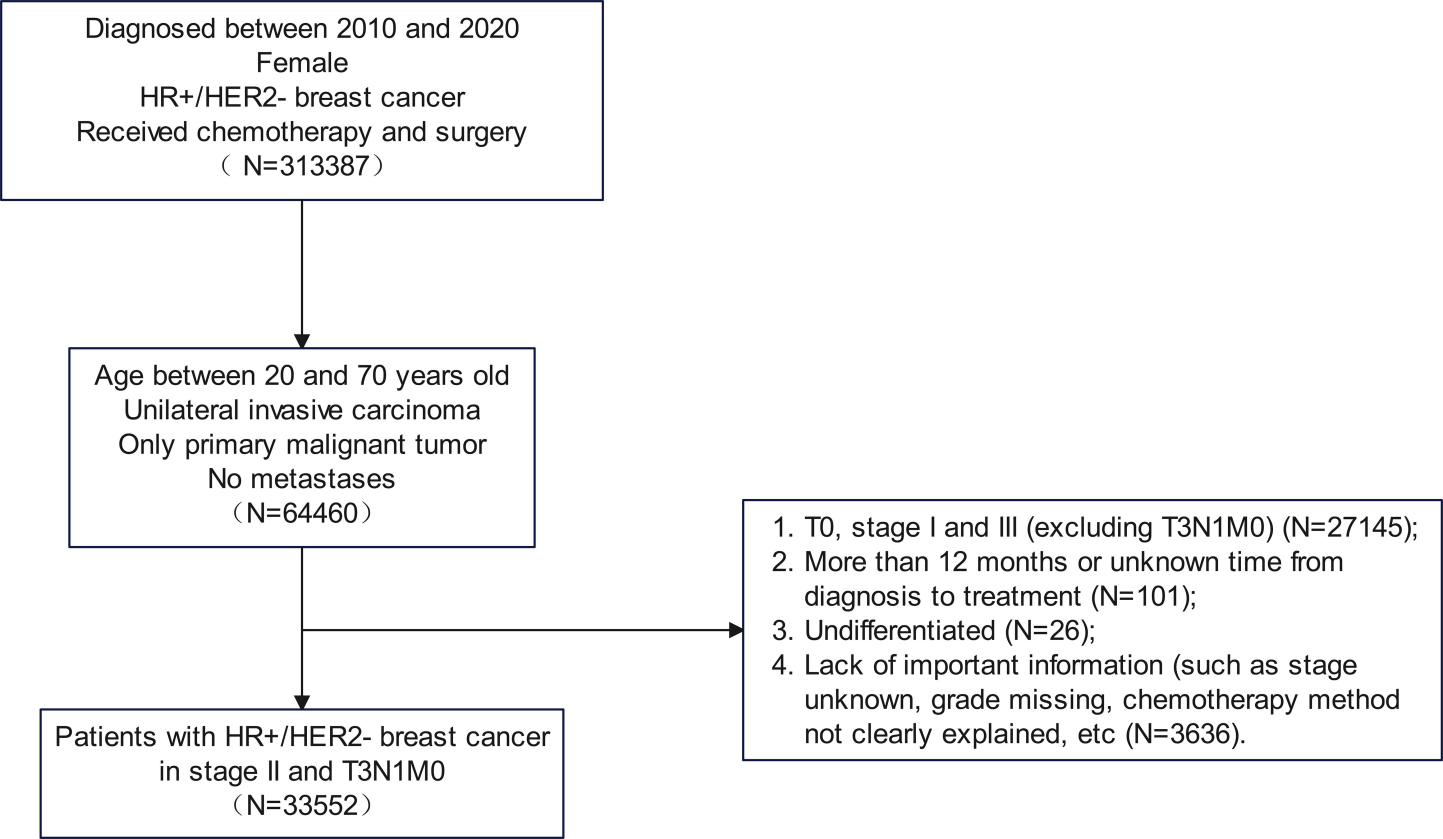
The flowchart of patients.

Patients were included if they met the following criteria: 1) female patients diagnosed with unilateral invasive HR+/HER2- breast cancer; 2) aged 20–70 years; 3) underwent both chemotherapy and surgery; 4) absence of distant metastasis at diagnosis; and 5) breast cancer as the sole primary malignancy. Patients were excluded based on the following criteria: 1) clinical stage I or III disease (except T3N1M0); 2) grade IV (undifferentiated) tumors; 3) interval of >12 months or unknown duration from diagnosis to treatment initiation; and 4) missing critical clinicopathologic data. The patient selection process is illustrated in **Figure 1**.

Given the retrospective nature of this study and the use of de-identified data, informed consent was waived. All procedures were conducted in compliance with the Declaration of Helsinki. This study was reported in accordance with the Strengthening the Reporting of Observational Studies in Epidemiology (STROBE) guidelines (17).

### Demographic and clinicopathologic information

The following variables were extracted from the SEER database: sex, age, race, TNM stage, histological type (classified as invasive ductal carcinoma [IDC], invasive lobular carcinoma [ILC], or other types), histological grade, type of surgery, sequence of chemotherapy, receipt of radiotherapy, response to NACT, ER and PR status, cause of death, survival time, and vital status. Based on the SEER Program Coding and Staging Manual 2021, surgical procedures were categorized as breast-conserving surgery (BCS), mastectomy, or reconstruction. Patients were classified into the NACT group or ACT group based on the “Chemotherapy recode” and “RX Summ--Systemic/Sur Seq” variables in the SEER database. Patients who received systemic therapy before surgery were classified as NACT, while those who received systemic therapy after surgery were classified as ACT.

The response to NACT was classified as complete response (CR), defined as the complete disappearance of the target lesion following chemotherapy; partial response (PR), defined as a ≥30% reduction in the longest diameter of the target lesion compared with baseline; or no response (NR), defined as <30% reduction or any increase in lesion size. For analytical purposes, CR and PR were combined and defined as objective response (OR), because a substantial proportion of response data in the SEER database were recorded as combined CR/PR categories without a reliable distinction between complete response and partial response.

### Outcomes

The primary endpoints of this study were OS and BCSS. OS was defined as the interval from the date of diagnosis to the date of death from any cause or the last follow-up, whichever occurred first. BCSS was defined as the interval from the date of diagnosis to the date of death attributed to breast cancer or the last follow-up, whichever occurred first. Follow-up duration was calculated from the date of diagnosis to the date of death or last follow-up, whichever occurred first.

### Statistical analyses

Continuous variables were presented as mean ± standard deviation (SD) for normally distributed data or median with interquartile range (IQR) for non-normally distributed data. Categorical variables were expressed as frequencies and percentages, and compared using the chi-square test. Normality was assessed using the Shapiro-Wilk test. Patients with missing data on key variables were excluded from the analysis (complete-case analysis).

Propensity score matching (PSM) was performed to reduce selection bias between the NACT and ACT groups. Propensity scores were calculated by multivariate logistic regression based on baseline variables, including age, T stage, N stage, histological type, grade, type of surgery, radiotherapy, race, ER and PR status. The NACT and ACT groups were matched at a ratio of 1:1 using the nearest neighbor matching method, with the caliper set at 0.02. The balance of baseline covariates before and after matching was evaluated using standardized mean differences (SMD), with an SMD < 0.1 indicating adequate balance.

Survival curves were generated using the Kaplan-Meier method, and the log-rank test was used to compare survival differences between groups. The 5-year OS and BCSS rates were estimated using the Kaplan-Meier method. Univariate Cox proportional hazards regression analysis was initially performed, and variables with P < 0.05 were subsequently included in the multivariate model. Cox proportional hazards regression models were used to estimate the effects of NACT on BCSS and OS, with hazard ratios (HRs) and 95% confidence intervals (CIs) reported. The proportional hazards assumption was verified using Schoenfeld residuals. Subgroup analyses were performed and forest plots were constructed to identify subgroup characteristics that might benefit from NACT. Subgroup analyses were conducted according to the following variables: age (<50 vs ≥50 years), race (White, Black, and Others), histological type (IDC, ILC, and others), histological grade (I, II, and III), T stage (T1, T2, and T3), N stage (N0 and N1), ER status (positive vs negative), and PR status (positive vs negative).

All analyses were performed using R version 4.4.1. *p* < 0.05 for the two-sided test was considered statistically significant.

## Results

### Baseline characteristics of the total population before and after PSM

A total of 33,552 patients meeting the eligibility criteria were included in this study, comprising 3,661 in the NACT group and 29,891 in the ACT group. The median follow-up duration was 62 months overall, with 52 months in the NACT group and 63 months in the ACT group. The objective response (OR) rate in the NACT group was 61.76%.

**Figure 2:**
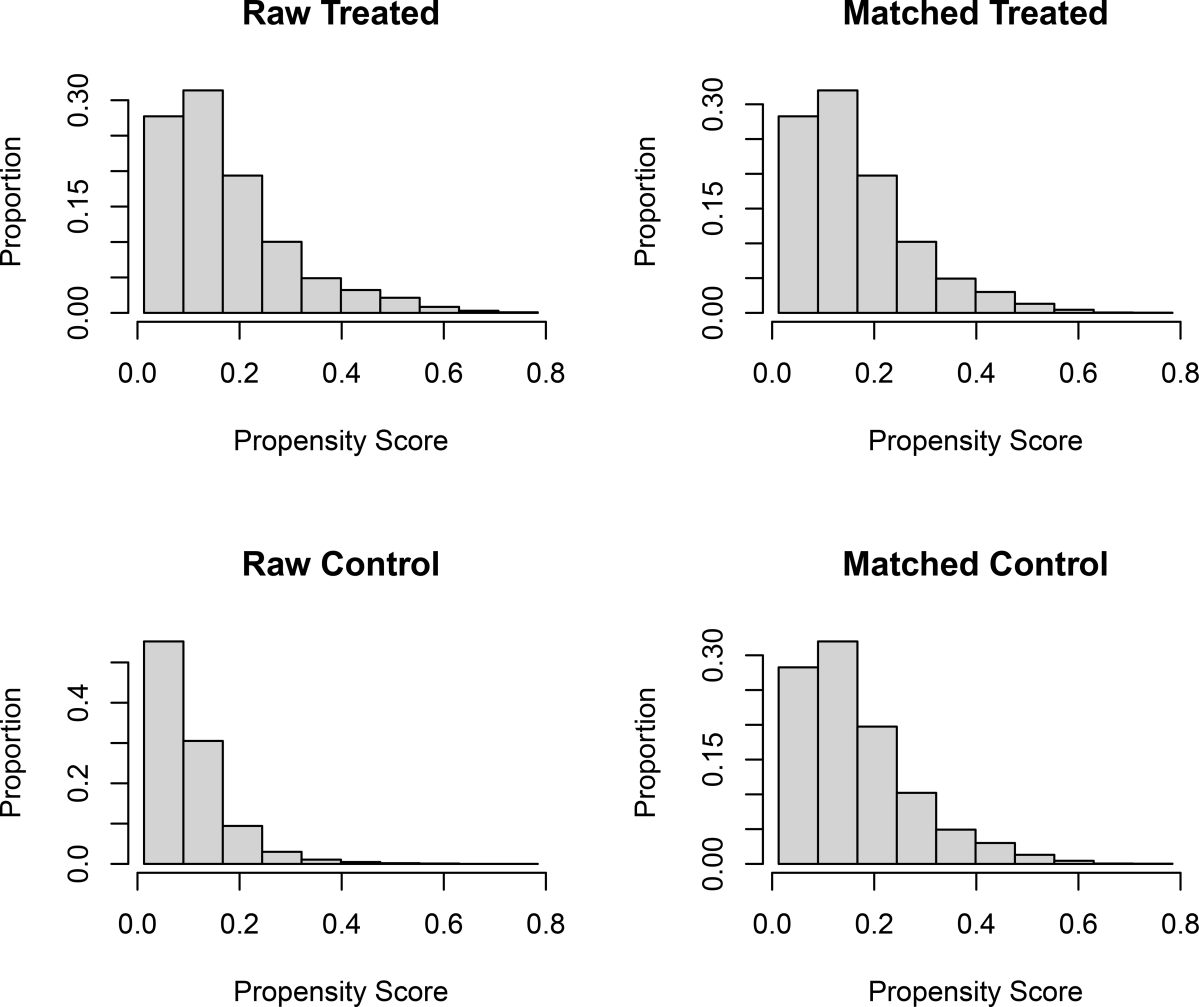
Propensity score equalization results.

Before PSM, significant differences in baseline characteristics were observed between the two groups. Compared with the ACT group, patients in the NACT group were more likely to be Black (14.8% vs 10.8%, P < 0.001), have grade III tumors (49.9% vs 35.2%, P < 0.001), T3 stage (23.8% vs 8.7%, P < 0.001), N0 status (35.0% vs 32.4%, P = 0.001), invasive ductal carcinoma (IDC) histology (83.3% vs 78.4%, P < 0.001), ER-negative status (7.1% vs 1.7%, P < 0.001), and PR-negative status (26.5% vs 14.4%, P < 0.001). Conversely, patients in the ACT group were more likely to be aged ≥50 years (60.7% vs 48.9%, P < 0.001), undergo BCS (48.9% vs 38.2%, P < 0.001), and receive radiotherapy (62.8% vs 57.0%, P < 0.001). To minimize the impact of baseline imbalances on outcome assessment, PSM was performed, yielding 7,182 matched patients (3,591 in each group). The propensity score distribution before and after matching is visualized in **Figure 2**. After matching, all baseline covariates were well-balanced between the two groups, with all standardized mean differences (SMD) < 0.1 (**Table 1**).

**Table 1:** Baseline variables before and after PSM for patients receiving neoadjuvant or adjuvant chemotherapy.

Variable	Before matching		*p*	After matching		*p*
	ACT group (N = 29891)	NACT group (N = 3661)		ACT group (N = 3591)	NACT group (N = 3591)	
Months from diagnosis to treatment, median (IQR)	1 (1-2)	1 (1-2)	—	1 (1-2)	1 (1-2)	—
Survival months, median (IQR)	63 (32-95)	52 (24-86)	—	59 (29-92)	52 (24-86)	—
Grade, n (%)			<0.001			0.994
I	4141 (13.9)	290 (7.9)		288 (8.0)	290 (8.1)	
II	15230 (51.0)	1545 (42.2)		1536 (42.8)	1538 (42.8)	
III	10520 (35.2)	1826 (49.9)		1767 (49.2)	1763 (49.1)	
T stage, n (%)			<0.001			0.704
T1	7792 (26.1)	427 (11.7)		427 (11.9)	427 (11.9)	
T2	19504 (65.3)	2361 (64.5)		2375 (66.1)	2346 (65.3)	
T3	2595 (8.7)	873 (23.8)		789 (22.0)	818 (22.8)	
N stage, n (%)			0.001			0.552
N0	9680 (32.4)	1282 (35.0)		1235 (34.4)	1260 (35.1)	
N1	20211 (67.6)	2379 (65.0)		2356 (65.6)	2331 (64.9)	
Histological type, n (%)			<0.001			0.637
IDC	23426 (78.4)	3051 (83.3)		2999 (83.5)	2984 (83.1)	
IDC/ILC	2143 (7.2)	175 (4.8)		162 (4.5)	175 (4.9)	
ILC	3418 (11.4)	297 (8.1)		310 (8.6)	297 (8.3)	
Other	904 (3.0)	138 (3.8)		120 (3.3)	135 (3.8)	
Surgical methods, n (%)			<0.001			0.925
BCS	14629 (48.9)	1398 (38.2)		1378 (38.4)	1390 (38.7)	
Mastectomy	9224 (30.9)	1381 (37.7)		1360 (37.9)	1344 (37.4)	
Reconstruction	6038 (20.2)	882 (24.1)		853 (23.8)	857 (23.9)	
Age, n (%)			<0.001			0.423
≤34 years	1089 (3.6)	306 (8.4)		257 (7.2)	286 (8.0)	
35–49 years	10654 (35.6)	1563 (42.7)		1546 (43.1)	1524 (42.4)	
≥50 years	18148 (60.7)	1792 (48.9)		1788 (49.8)	1781 (49.6)	
Radiation, n (%)			<0.001			0.886
No	11110 (37.2)	1574 (43.0)		1538 (42.8)	1531 (42.6)	
Yes	18781 (62.8)	2087 (57.0)		2053 (57.2)	2060 (57.4)	
Race, n (%)			<0.001			0.819
Other*	3882 (13.0)	441 (12.0)		424 (11.8)	434 (12.1)	
Black	3217 (10.8)	541 (14.8)		536 (14.9)	519 (14.5)	
White	22792 (76.3)	2679 (73.2)		2631 (73.3)	2638 (73.5)	
ER status, n (%)			<0.001			0.337
Negative	519 (1.7)	259 (7.1)		242 (6.7)	221 (6.2)	
Positive	29372 (98.3)	3402 (92.9)		3349 (93.3)	3370 (93.8)	
PR status, n (%)			<0.001			0.914
Negative	4299 (14.4)	969 (26.5)		934 (26.0)	939 (26.1)	
Positive	25592 (85.6)	2692 (73.5)		2657 (74.0)	2652 (73.9)	
Response to NACT, n (%)			—			—
NR	—	197 (5.4)		—	191 (5.3)	
OR	—	2261 (61.8)		—	2215 (61.7)	
Unknown	—	1203 (32.9)		—	1185 (33.0)	

PSM, propensity score matching; ACT, adjuvant chemotherapy; NACT, neoadjuvant chemotherapy; SMD, standardized mean difference; IQR, interquartile range; IDC, invasive ductal carcinoma; ILC, invasive lobular carcinoma; BCS, breast-conserving surgery; ER, estrogen receptor; PR, progesterone receptor; NR, no response; OR, objective response. “—” indicates not applicable. SMD < 0.1 indicates adequate balance between groups.

*American Indian/AK Native, Asian/Pacific Islander.

### Survival analysis before and after PSM

**Figure 3:**
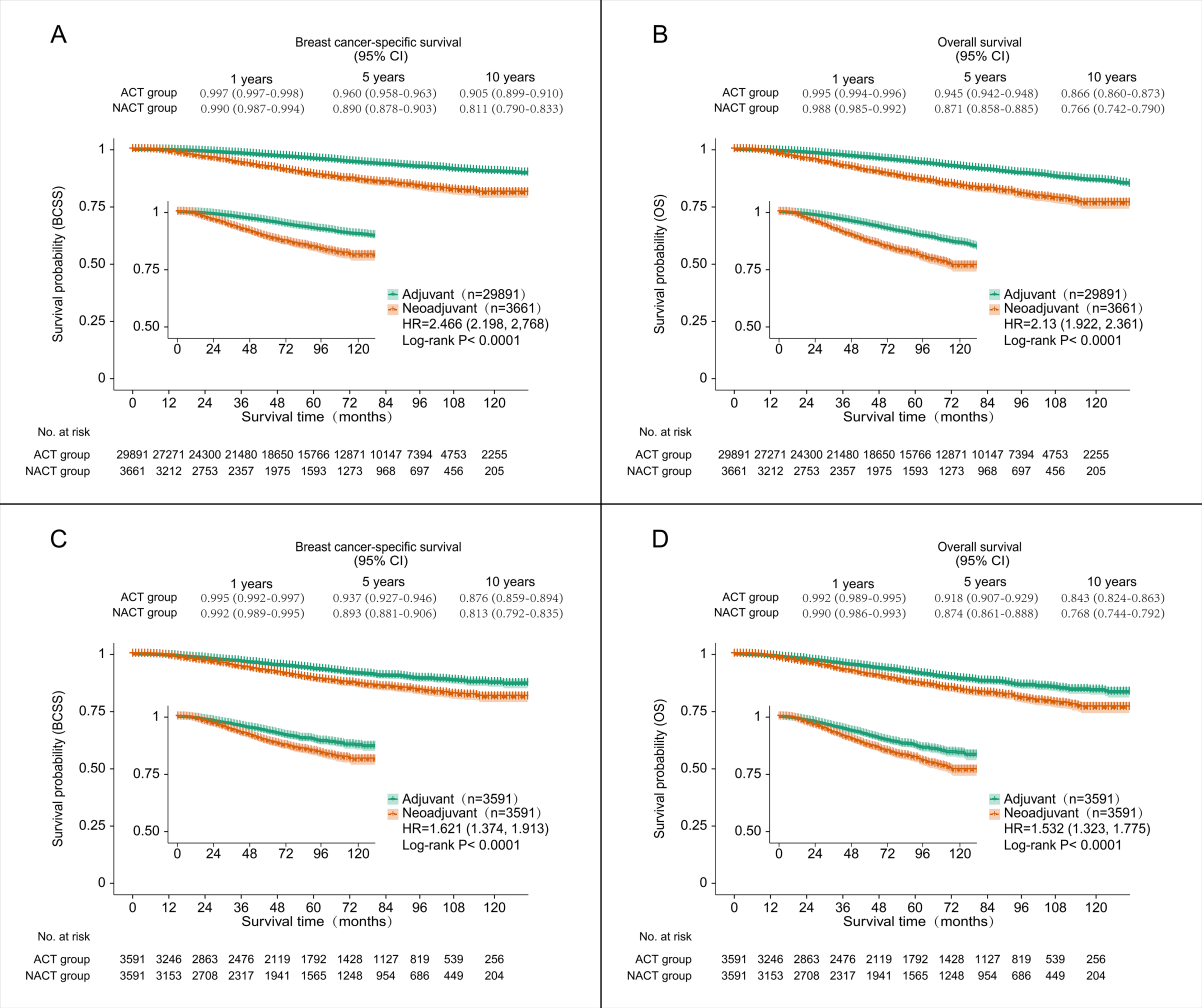
Kaplan-Meier survival curves for BCSS and OS in patients treated with ACT and those treated with NACT before and after PSM. **(A)** Kaplan-Meier survival curves for BCSS before PSM. **(B)** Kaplan-Meier survival curves for OS before PSM. **(C)** Kaplan-Meier survival curves for BCSS after PSM. **(D)** Kaplan-Meier survival curves for OS after PSM. BCSS, breast cancer-specific survival; OS, overall survival; ACT, adjuvant chemotherapy; NACT, neoadjuvant chemotherapy; PSM, propensity score matching.

Before PSM, a total of 2,435 deaths (7.26%) occurred in the overall population, including 446 (12.18%) in the NACT group and 1,989 (6.65%) in the ACT group. Among these, 1,767 deaths were attributed to breast cancer, with 364 (9.94%) in the NACT group and 1,403 (4.69%) in the ACT group. Log-rank test showed significant differences in OS and BCSS between the two groups, with *p* < 0.0001 in both groups (**Figures 3A, B**).

After PSM, among the 7,182 matched patients, 734 deaths (10.22%) were recorded, comprising 428 (11.92%) in the NACT group and 306 (8.52%) in the ACT group. Breast cancer-specific deaths totaled 583 (8.12%), with 348 (9.69%) in the NACT group and 235 (6.54%) in the ACT group. Log-rank test showed significant differences in OS and BCSS between the two groups, with *p* < 0.0001 in both groups (**Figures 3C, D**).

### Multivariate cox regression analysis after PSM

Univariate Cox regression analysis was initially conducted in the matched cohort, and variables with *p* < 0.05 were subsequently incorporated into the multivariate Cox regression model. After adjusting for histological grade, T stage, N stage, type of surgery, age, race, response to NACT, and ER/PR status, multivariate analysis demonstrated that patients with no response (NR) to NACT had significantly inferior BCSS (HR = 3.36, 95% CI: 2.28–4.96, P < 0.001) and OS (HR = 3.30, 95% CI: 2.35–4.64, P < 0.001) compared with the ACT group. Notably, even among patients who achieved OR following NACT, both BCSS (HR = 1.42, 95% CI: 1.17–1.73, P < 0.001) and OS (HR = 1.31, 95% CI: 1.10–1.57, P = 0.002) remained inferior to those in the ACT group. Additional independent prognostic factors associated with worse outcomes included higher histological grade, larger tumor size, axillary lymph node metastasis, and ER-negative or PR-negative status. Patients who underwent mastectomy exhibited worse prognosis compared with those who received BCS. Black patients demonstrated inferior survival outcomes compared with White and other patients (**Table 2**).

**Table 2:** Univariate and multivariate Cox regression analysis on BCSS and OS in matched populations.

Variable	Univariate				Multivariate			
	BCSS HR (95% CI)	*p*	OS HR (95% CI)	*p*	BCSS HR (95% CI)	*p*	OS HR (95% CI)	*p*
Grade								
I	Ref		Ref		Ref		Ref	
II	2.19 (1.33-3.61)	0.002	1.52 (1.05-2.20)	0.026	2.10 (1.28-3.46)	0.003	1.46 (1.01-2.12)	0.043
III	4.37 (2.69-7.11)	<0.001	2.82 (1.97-4.03)	<0.001	4.21 (2.57-6.89)	<0.001	2.72 (1.88-3.92)	<0.001
T stage								
T1	Ref		Ref		Ref		Ref	
T2	1.69 (1.22-2.36)	0.002	1.67 (1.25-2.24)	<0.001	1.86 (1.33-2.60)	<0.001	1.81 (1.35-2.44)	<0.001
T3	2.54 (1.80-3.59)	<0.001	2.46 (1.81-3.34)	<0.001	2.70 (1.90-3.84)	<0.001	2.56 (1.87-3.50)	<0.001
N stage								
N0	Ref		Ref		Ref		Ref	
N1	1.60 (1.33-1.93)	<0.001	1.42 (1.21-1.68)	<0.001	1.82 (1.50-2.21)	<0.001	1.60 (1.35-1.89)	<0.001
Histological type								
IDC	Ref		Ref		—		—	
IDC/ILC	0.84 (0.57-1.23)	0.373	0.71 (0.49-1.03)	0.071	—		—	
ILC	0.80 (0.59-1.09)	0.153	0.87 (0.67-1.13)	0.304	—		—	
Other	1.01 (0.66-1.55)	0.962	1.02 (0.70-1.49)	0.909	—		—	
Surgical methods								
BCS	Ref		Ref		Ref		Ref	
Mastectomy	1.78 (1.47-2.15)	<0.001	1.67 (1.41-1.97)	<0.001	1.71 (1.40-2.08)	<0.001	1.62 (1.36-1.92)	<0.001
Reconstruction	1.24 (0.99-1.56)	0.065	1.04 (0.85-1.29)	0.681	1.25 (0.99-1.59)	0.061	1.09 (0.88-1.35)	0.419
Age								
≤34 years	Ref		Ref		Ref		Ref	
35–49 years	0.73 (0.54-0.99)	0.045	0.79 (0.59-1.05)	0.107	0.90 (0.66-1.22)	0.493	0.94 (0.70-1.26)	0.69
≥50 years	0.90 (0.67-1.21)	0.499	1.10 (0.83-1.46)	0.501	1.04 (0.77-1.41)	0.796	1.22 (0.92-1.62)	0.173
Radiation								
No	Ref		Ref		—		—	
Yes	1.14 (0.97-1.35)	0.117	1.03 (0.89-1.19)	0.704	—		—	
Race								
Black	Ref		Ref		Ref		Ref	
Other*	0.45 (0.32-0.62)	<0.001	0.43 (0.32-0.58)	<0.001	0.51 (0.36-0.72)	<0.001	0.50 (0.37-0.67)	<0.001
White	0.63 (0.51-0.76)	<0.001	0.60 (0.50-0.71)	<0.001	0.70 (0.57-0.86)	<0.001	0.67 (0.56-0.80)	<0.001
ER status								
Negative	Ref		Ref		Ref		Ref	
Positive	0.72 (0.54-0.96)	0.024	0.77 (0.59-1.00)	0.052	0.65 (0.48-0.89)	0.008	0.71 (0.53-0.95)	0.021
PR status								
Negative	Ref		Ref		Ref		Ref	
Positive	0.57 (0.49-0.68)	<0.001	0.59 (0.51-0.69)	<0.001	0.67 (0.56-0.81)	<0.001	0.69 (0.58-0.81)	<0.001
Response to NACT								
ACT	Ref		Ref		Ref		Ref	
NR	3.29 (2.23-4.83)	<0.001	3.32 (2.37-4.65)	<0.001	3.36 (2.28-4.96)	<0.001	3.30 (2.35-4.64)	<0.001
OR	1.38 (1.14-1.68)	0.001	1.27 (1.07-1.52)	0.007	1.42 (1.17-1.73)	<0.001	1.31 (1.10-1.57)	0.002
Unknown	1.84 (1.50-2.27)	<0.001	1.77 (1.47-2.13)	<0.001	1.96 (1.59-2.42)	<0.001	1.86 (1.54-2.24)	<0.001

BCSS, breast cancer-specific survival; OS, overall survival; HR, hazard ratio; CI, confidence interval; Ref, reference; IDC, invasive ductal carcinoma; ILC, invasive lobular carcinoma; BCS, breast-conserving surgery; ER, estrogen receptor; PR, progesterone receptor; NACT, neoadjuvant chemotherapy; ACT, adjuvant chemotherapy; NR, no response; OR, objective response. “—” indicates variables not included in the multivariate model.

*American Indian/AK Native, Asian/Pacific Islander.

### Subgroup analysis

**Figure 4:**
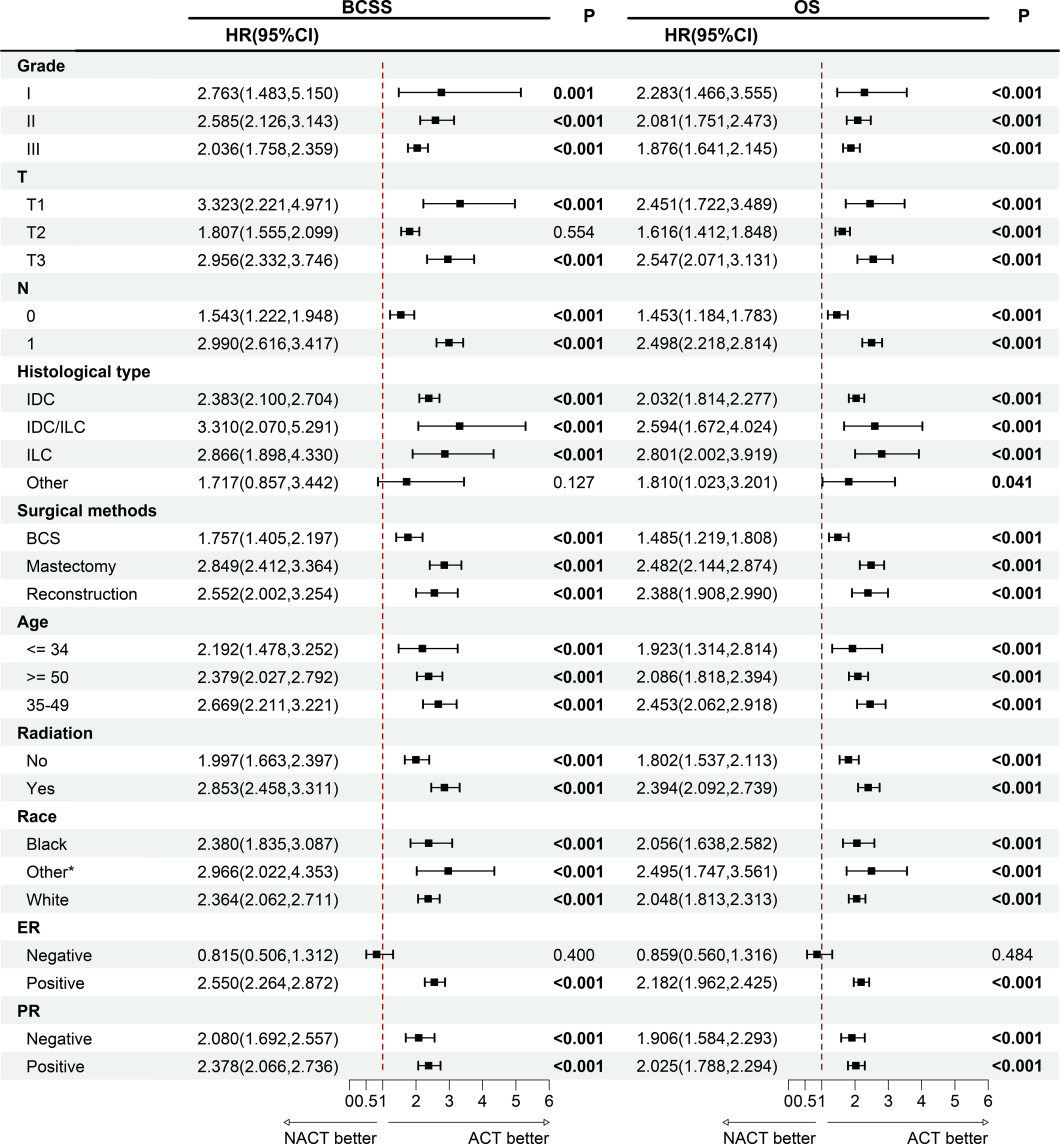
Survival outcomes of patients who received NACT and ACT in each subgroup of patients. *American Indian/AK Native, Asian/Pacific Islander. BCSS, breast cancer-specific survival; OS, overall survival; HR, hazard ratio; CI, confidence interval; IDC, invasive ductal carcinoma; ILC, invasive lobular carcinoma; BCS, breast conserving surgery; ER, estrogen receptor; PR, progesterone receptor; NACT, neoadjuvant chemotherapy; ACT, adjuvant chemotherapy.

To further explore the factors contributing to the inferior outcomes associated with NACT, subgroup analysis was conducted and forest plots were generated. In most subgroups except ER-negative, the NACT group showed a higher risk of total and breast cancer-specific mortality. NACT was associated with worse OS and BCSS in most subgroups, except other histological types and T2 subgroups. There were statistically significant differences in these results (**Figure 4**).

## Discussion

In this large retrospective cohort study using the SEER database, patients with operable HR+/HER2- breast cancer at stage II or T3N1M0 who received NACT had inferior OS and BCSS compared with those who received ACT. Notably, this survival disadvantage persisted even among patients who achieved OR to NACT. These findings remained robust after PSM to balance potential confounding factors between the two groups.

At present, the indication for NACT in patients with early-stage operable breast cancer remains a controversial issue. High-quality studies specifically evaluating the effects of NACT versus ACT on long-term survival outcomes in patients with HR+/HER2- breast cancer at stage II and T3N1M0 are lacking, and guidelines on the indications for NACT are inconsistent (5, 11–14). Consequently, considerable heterogeneity exists in clinical decision-making regarding chemotherapy sequencing. The results of previous classical randomized controlled trials and meta-analyses demonstrated no significant difference in survival outcomes between NACT and ACT (1, 2). However, these studies were not specifically designed for patients with HR+/HER2- breast cancer at stage II or T3N1M0, outcomes by molecular subtype, and did not account for differences in response to NACT or advances in contemporary diagnostic and treatment strategies. The present findings are consistent with the study by Shang et al. (15), which reported inferior survival outcomes with NACT in T2N1M0 HR+/HER2- breast cancer patients using the SEER database. Similarly, a recent large-scale SEER-based study demonstrated that NACT was associated with poorer OS and BCSS compared with ACT in stage II-III HR+/HER2- breast cancer patients, with 5-year OS of 83.7% versus 89.6% after PSM (18). However, the current results contrast with the Swedish population-based study, which demonstrated comparable outcomes between NACT and ACT in HR+/HER2- breast cancer (16). This discrepancy may be attributed to differences in patient selection criteria, healthcare systems, treatment protocols, and the availability of adjuvant therapies between the United States and Sweden.

Several mechanisms may explain the inferior outcomes associated with NACT in this population. HR+/HER2- breast cancers are generally characterized by lower proliferation rates and are inherently less sensitive to cytotoxic chemotherapy compared with triple-negative and HER2-positive subtypes (19, 20). A multicenter retrospective analysis reported that the pCR rate in luminal breast cancer following NACT was only 11.3%, with luminal A-like tumors achieving pCR in merely 5.3% of cases (20). This is substantially lower than the pCR rates observed in triple-negative breast cancer (30-40%) and HER2-positive breast cancer (50-70%). Moreover, unlike in triple-negative and HER2-positive breast cancer, pCR in HR+/HER2- tumors does not consistently translate into improved long-term survival outcomes (6, 10). In addition to the limited chemosensitivity, the inherent delay in definitive surgery associated with NACT may increase the risk of disease progression, particularly in tumors that respond poorly to chemotherapy, and this effect may be more pronounced in patients with stage IIA-IIIA breast cancer (21, 22). Previous studies have also demonstrated that tumors in the NACT group were generally staged later and less differentiated compared with the ACT group, contributing to poorer survival outcomes (23). A recent meta-analysis demonstrated that surgical delays exceeding 8 weeks following NACT were associated with worse OS and recurrence-free survival (24). Although the NACT duration typically ranges from 4 to 6 months, the cumulative effect of delayed surgery in poorly responsive tumors may contribute to inferior outcomes. Clinical evaluation after NACT can reflect tumor chemosensitivity, and the results of Cox proportional hazards regression in the present study demonstrated that patients with clinical evaluation of no response (NR) after NACT had a higher risk of death and breast cancer-specific death compared with those who achieved OR. However, even among patients who achieved OR, long-term outcomes were not superior to those of the ACT group, suggesting that clinical response alone may not adequately capture the biological behavior of HR+/HER2- tumors. It should also be noted that the SEER database lacks information on endocrine therapy use, as well as detailed information on chemotherapy regimens, doses, and cycles, all of which are highly relevant to the prognosis of HR+/HER2- breast cancer. Differences in the timing, duration, and adherence to adjuvant endocrine therapy between the NACT and ACT groups could not be accounted for and may have influenced survival outcomes.

Nowadays, a portion of HR+/HER2- breast cancer patients can be exempted from chemotherapy after surgery by multigene testing (25, 26). In Chinese clinical practice, patients with HR+/HER2- breast cancer typically do not undergo multigene testing to assess chemotherapy sensitivity before surgery, as this approach is not supported by current evidence-based medicine guidelines. Previous studies have correlated genetic scores with pathologic response, finding that high OncotypeDX recurrence scores or high MammaPrint scores correlate with higher pCR rate (27). Therefore, it remains to be investigated whether preoperative multigene testing could be used to predict chemotherapy sensitivity in HR+/HER2- breast cancer and guide the selection between NACT, ACT, or exemption from chemotherapy with a focus on intensive endocrine therapy. This hypothesis warrants further investigation in prospective studies. Furthermore, neoadjuvant endocrine therapy (NET) has emerged as an alternative approach for postmenopausal patients with HR+/HER2- breast cancer (28). NET may offer comparable clinical response rates with potentially fewer adverse effects compared with NACT in this molecularly defined subgroup. Future studies should explore whether NET could serve as a preferred neoadjuvant strategy for selected HR+/HER2- breast cancer patients.

The present study has several strengths. The SEER database provided a large sample size and adequate follow-up duration, which strengthens the credibility of the findings. The use of PSM helped to balance baseline characteristics between the NACT and ACT groups, reducing the impact of selection bias. Additionally, subgroup analyses were performed to identify patient populations that may or may not benefit from NACT, providing clinically relevant information for treatment decision-making. However, this study also has several limitations that should be acknowledged. As a retrospective analysis of the SEER database, inherent selection bias cannot be entirely eliminated despite PSM adjustment. Although PSM balanced the observed baseline variables, residual confounding and confounding by indication may still be present, because patients selected for NACT may have had more aggressive tumor biology, greater tumor burden, or other unfavorable clinical characteristics that were not captured in the SEER database. Patients selected for NACT may have had more aggressive tumor characteristics or larger tumor burden at presentation, which could confound the survival comparison. In addition, the SEER database also lacks information on Ki-67 levels, quantitative ER and PR expression, endocrine therapy use, detailed chemotherapy regimens, doses and cycles, targeted therapy administration, and several other treatment-related variables, all of which may influence survival outcomes. Data on disease recurrence and metastasis were unavailable, precluding analyses of disease-free survival or recurrence-free survival as additional endpoints. Other unmeasured confounders, such as patient performance status, comorbidities, treatment adherence, and socioeconomic factors, could not be accounted for either. Furthermore, the generalizability of these findings to non-U.S. populations remains uncertain given potential differences in treatment patterns and healthcare systems. Future prospective studies incorporating comprehensive molecular profiling, detailed treatment information, and long-term follow-up data are warranted to validate these findings and refine treatment selection criteria for patients with HR+/HER2- breast cancer.

From a clinical perspective, these findings suggest that the decision to administer NACT in patients with operable HR+/HER2- breast cancer should be carefully weighed against the potential survival disadvantage. In patients where surgical downstaging is not required, proceeding directly to surgery followed by ACT may be the preferred approach. However, for patients requiring tumor downstaging to achieve breast conservation or in cases where *in vivo* assessment of tumor chemosensitivity is desired, NACT remains a valid option with appropriate patient counseling regarding the potential risks.

In summary, after adjustment for baseline characteristics using SEER database, NACT was associated with inferior long-term outcomes compared with ACT in patients with operable HR+/HER2- breast cancer at stage II or T3N1M0. This finding requires validation in prospective multicenter studies with comprehensive data collection on treatment regimens and molecular characteristics. Nevertheless, these findings suggest that NACT may not be the optimal treatment strategy for some patients with operable HR+/HER2- breast cancer. Caution should be exercised when selecting NACT for this patient population.

## Data Availability

Publicly available datasets were analyzed in this study. This data can be found here: The data underlying this article are available in the SEER database, at https://seer.cancer.gov/.
